# Correction: Sundaram et al. Effects of Intermediate Frequency (150 kHz) Electromagnetic Radiation on the Vital Organs of Female Sprague Dawley Rats. *Biology* 2023, *12*, 310

**DOI:** 10.3390/biology13030181

**Published:** 2024-03-12

**Authors:** Venkatesan Sundaram, Stephanie Mohammed, Brian N. Cockburn, M. R. Srinivasan, Chalapathi R. Adidam Venkata, Jenelle Johnson, Lester Gilkes, Kegan Romelle Jones, Nikolay Zyuzikov

**Affiliations:** 1 Department of Basic Veterinary Sciences, School of Veterinary Medicine, Faculty of Medical Sciences, The University of the West Indies, St. Augustine 999183, Trinidad and Tobago; venkatesan.sundaram@sta.uwi.edu (V.S.); lester.gilkes@sta.uwi.edu (L.G.); 2 Department of Physics, Faculty of Science and Technology, The University of the West Indies, St. Augustine 999183, Trinidad and Tobago; stephanie.mohammed@sta.uwi.edu (S.M.); nikolay.zyuzikov@sta.uwi.edu (N.Z.); 3 Department of Life Sciences, Faculty of Science and Technology, The University of the West Indies, St. Augustine 999183, Trinidad and Tobago; brian.cockburn@sta.uwi.edu; 4 Laboratory Animal Medicine Unit, Directorate of Centre for Animal Health Studies, Tamil Nadu Veterinary and Animal Sciences University, Chennai 600016, Tamil Nadu, India; seenubioinfo@gmail.com; 5 Department of Paraclinical Sciences, Faculty of Medical Sciences, The University of the West Indies, St. Augustine 999183, Trinidad and Tobago; chalapathi.rao@sta.uwi.edu; 6 Department of Clinical Veterinary Sciences, School of Veterinary Medicine, Faculty of Medical Sciences, The University of the West Indies, St. Augustine 999183, Trinidad and Tobago; jenelle.johnson2@sta.uwi.edu

## Email Correction

In the original publication [1], there was an error regarding the email address of Lester Gilkes. The email address changed from lester.gikes@sta.uwi.edu to lester.gilkes@sta.uwi.edu.

## Error in Figure

The original publication contained an error in “Figure 7. The effect of 150 kHz IF EMR on the histopathology of the stomach, intestine (jejunum), and skin of rats in the control and EMR groups after 2 months of whole-body exposure” [1]. The images representing the intestine in Figure 7 mistakenly included two different pictures taken from the same animal (belonging to the control group) to depict both the control and experimental (EMR) groups. This oversight occurred unintentionally while managing numerous images during the compilation process. The correct intestinal images for the experimental (EMR) group have now been substituted in Figure 7. The corrected “Figure 7. The effect of 150 kHz IF EMR on the histopathology of the stomach, intestine (jejunum), and skin of rats in the control and EMR groups after 2 months of whole-body exposure” is provided below. The authors state that the scientific conclusions remain unaffected, as these images serve as representative figures only, and the results and conclusions were drawn from histological observations conducted on all animals.

The authors apologize for any inconvenience caused and state that the scientific conclusions are unaffected. This correction was approved by the Academic Editor. The original publication has also been updated.

## Figures and Tables

**Figure 7:**
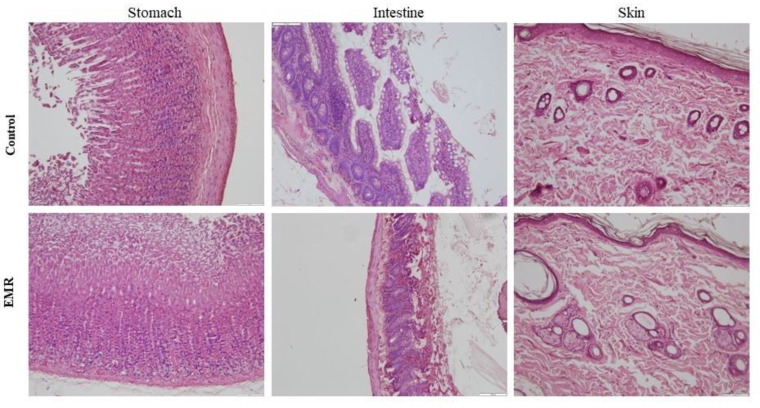
The effect of 150 kHz IF EMR on the histopathology of the stomach, intestine (jejunum), and skin of rats in the control and EMR groups after 2 months of whole-body exposure. The mucosal epithelium and glands of the stomach appeared normal. The lamina propria appeared to be healthy. The intestine showed a typical mucosa with enterocytes and goblet cells lining the villi. The epidermis and dermis of the skin appeared normal with typical glands (H&E × 200).
